# The Cell Killing Mechanisms of Hydroxyurea

**DOI:** 10.3390/genes7110099

**Published:** 2016-11-17

**Authors:** Amanpreet Singh, Yong-Jie Xu

**Affiliations:** 1 Department of Pharmacology and Toxicology, Boonshoft School of Medicine, Wright State University, Dayton, OH 45435, USA; singh.73@wright.edu; 2 Wadsworth Center, NYSDOH, 120 New Scotland Ave., Albany, NY 12208, USA

**Keywords:** hydroxyurea, ribonucleotide reductase, oxidative stress, cytokinesis arrest, DNA replication checkpoint, cell cycle

## Abstract

Hydroxyurea is a well-established inhibitor of ribonucleotide reductase that has a long history of scientific interest and clinical use for the treatment of neoplastic and non-neoplastic diseases. It is currently the staple drug for the management of sickle cell anemia and chronic myeloproliferative disorders. Due to its reversible inhibitory effect on DNA replication in various organisms, hydroxyurea is also commonly used in laboratories for cell cycle synchronization or generating replication stress. However, incubation with high concentrations or prolonged treatment with low doses of hydroxyurea can result in cell death and the DNA damage generated at arrested replication forks is generally believed to be the direct cause. Recent studies in multiple model organisms have shown that oxidative stress and several other mechanisms may contribute to the majority of the cytotoxic effect of hydroxyurea. This review aims to summarize the progress in our understanding of the cell-killing mechanisms of hydroxyurea, which may provide new insights towards the improvement of chemotherapies that employ this agent.

## 1. Introduction

**Figure 1:**
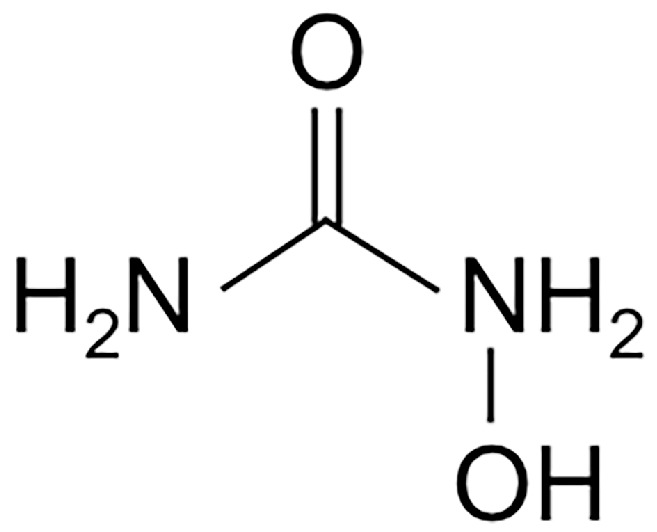
Hydroxyurea (HU).

Hydroxyurea (HU, also called hydroxycarbamide, see Figure 1) is a non-alkylating antineoplastic and antiviral agent that has been used for a variety of conditions in the disciplines of hematology, oncology, infectious disease and dermatology. It was first synthesized over a century ago in 1869 [1], but it was not until ~60 years later in 1928 that the biological effects of this simple antimetabolite compound on blood cells in rabbits were reported [2]. A large-scale drug screen carried out in the 1960s showed that it has anti-tumor activities, which revived interest in HU as a potential antineoplastic drug [3,4]. Subsequent studies showed that it could be used to treat several types of solid tumors and myeloproliferative disorders. The therapeutic spectrum for HU was also expanded to include various infectious diseases such as the human immunodeficiency virus [5,6,7,8,9]. Some earlier studies also reported that HU could be successfully used for the treatment of psoriasis, particularly in cases that have not responded to other treatment [10,11]. Although newer and more efficient agents have replaced HU in certain instances, as an established, reliable and well-tolerable small molecule drug for multiple neoplastic and non-neoplastic diseases, it is still being used in clinics. Currently, it serves as the staple drug for the treatment of sickle cell anemia and chronic myeloproliferative disorders [12,13] and is listed as an “essential medicine” by the World Health Organization [14].

HU is an inhibitor of DNA synthesis in many organisms and in cell culture systems [15,16]. As a result, HU is mainly active in the S-phase of the cell cycle and because of the easy reversibility of its action, HU has been commonly used in laboratories as a synchronizing agent in cell cultures. HU has been shown to induce chromosome damage in various organisms and is also cytotoxic depending on the concentration that is used, the duration of exposure, and the sensitivity of the cell lines [17]. HU can also cross the placenta and is teratogenic in animals [17,18]. Thus, the DNA damage such as the strand breaks caused by inhibition of DNA synthesis is generally believed to be responsible for its cytotoxicity, the anti-neoplastic activity and the teratogenic effects. However, the reversible effect of HU on DNA replication suggests that it is a cytostatic agent, and, in addition to the DNA damage, its cytotoxic effects may involve a more complex mechanism. Furthermore, the genetic backgrounds in most of the mammalian cell lines that were used in earlier studies are unknown, and thus the previously reported cytotoxic effects and the underlying mechanisms need to be reconsidered more carefully. Recent studies in several model organisms with defined genetic backgrounds showed that HU also generates oxidative stress and induces cytokinesis arrest in certain mutant cells [19,20,21,22,23,24], which likely contributes to the majority of the cell-killing and thus the therapeutic effects. The benefits of HU for the treatment of sickle cell anemia are likely the increased production of fetal hemoglobin via nitric oxide production [25] and the decreased adherence of red blood cells to vascular endothelial cells [26]. The effectiveness for management of refractory psoriasis is likely due to its inhibitory effect on epithelial proliferation, which restores the patients’ thickened epidermis to a more normal appearance [10]. Interestingly, endogenous HU has been found in the plasma and various tissues of many animal groups [27], including humans [28], which is likely produced by arginase from the intermediate of nitric oxide synthesis pathway hydroxyarginine. Because the concentrations of endogenous HU vary by as much as 25 folds between tissues, and the concentrations in certain types of tissues are high enough to be effective against bacterial or viral infections, HU could also act as a natural defense agent. However, the exact function of the endogenous HU remains largely unknown. In the following, we will briefly review the action of HU on its primary target ribonucleotide reductase (RNR) and then summarize the recent research progress on the cell-killing mechanisms of this clinically important drug.

## 2. Inhibition of RNR and Other Potential Metalloenzymes

RNR is the well-established primary cellular target of HU [29,30]. This enzyme catalyzes the reduction of ribonucleoside diphosphates to their corresponding deoxyribonucleotides as the precursors for DNA replication and repair. RNRs are unique enzymes in that they all require a protein thiyl radical for catalysis. There are three classes of RNRs, which employ different mechanisms for the generation of the protein thiyl radical. Class I RNRs exist in mammals, plants, yeasts and prokaryotes. They contain two dissociable dimeric subunits termed R1 and R2 and require oxygen for the generation of a stable tyrosyl radical by a di-iron center in the smaller R2 subunit. During catalysis, the tyrosyl radical is continuously shuttled to a cysteine residue in the larger R1 subunit and generates the thiyl protein radical required for activation of the substrate [31]. Computer modeling showed that this path of radical transfer is ~35 Å long in class I reductases [32]. In class Ia RNRs, the redox-active cysteines of thioredoxin or glutaredoxin are the electron donors [15,30,33]. In addition to the catalytic site, the R1 subunit also contains allosteric sites for the regulation of RNR activity and specificity. Due to the allosteric regulation, all RNRs can provide an appropriate balance of the four deoxyribonucleotide triphosphate (dNTP) precursors for DNA synthesis [34]. Because of the essential function in DNA replication and repair, RNR is also highly regulated during the cell cycle and in response to DNA damage or perturbed DNA replication via multiple mechanisms [35,36,37,38].

HU inhibits RNR by directly reducing the diferric tyrosyl radical center in the smaller R2 subunit via one-electron transfer from the drug [16,29,39,40]. Since urea does not have such an effect [29], the –NH_2_-OH moiety of HU is the minimal structural requirement for the inhibitory effect. This conclusion is also supported by structure activity relationship studies [41,42,43]. Because the free radical catalysis mechanism is conserved among different RNRs from prokaryotes to higher eukaryotes, including mammals, HU has been proved to be active in many organisms. Free radicals are generally very reactive and short-lived. Therefore, few proteins utilize free radical chemistry. RNRs are remarkable in that they accomplish the catalysis through a complex radical storage and a long-range radical transfer mechanism. The tyrosyl radical in the R2 subunit is relatively stable. For example, the radical in *Escherichia coli* R2 can last for days at room temperature, although the same radical in mouse R2 needs to be continuously regenerated [44,45]. One explanation for the stability is that the tyrosyl radical is buried deep inside the protein. The three-dimensional structure of *E. coli* R2 protein showed that the radical is located more than 10Å from the closest surface within a hydrophobic pocket, an environment that is absolutely required for radical storage [46,47]. Because the crystal structure of a tetrameric RNR holoenzyme containing both R1 and R2 subunits has not been solved yet, the exact mechanism by which HU scavenges the tyrosyl radical and thus inhibits RNRs remains unclear. Since HU is a relatively small and simple molecule, it may penetrate into the R2 protein via small channels and directly access the tyrosyl radical [43,48]. Alternatively, HU scavenges the radical from the surface of RNR via a long-range electron transfer [44,48]. Since several bulkier and structurally unrelated compounds such as guanazole, pyrazoloimidazole (IMPY) and resveratrol [49,50] can also scavenge the tyrosyl radical, it is more likely that the radical is quenched via the long-range electron transfer mechanism. Kinetic studies of the HU scavenging reaction using purified *E. coli* R2 also support this mechanism [40,51]. Since the regulatory state of RNRs affects the radical stability and the radical in an active RNR holoenzyme is less stable in the presence of HU [48,51], HU may also exploit alternative sites along the electron-transfer path between the tyrosyl radical and the catalytic site on R1 through either direct or indirect access [48].

In addition to RNR, it has been reported that HU can target catalase in plant cells in vivo (see Table 1) [52]. HU can also suppress several other metalloenzymes in vitro such as carbonic anhydrase and matrix metalloproteases [53,54,55,56]. Because suppression of these metal enzymes occurs only in the presence of high concentrations of HU, whether HU targets these enzymes in vivo, particularly in the mutant cells with defects that can synergize with this HU effect, remains to be seen (see below).

## 3. S Phase Arrest, DNA Damage and the Checkpoint Response

Because RNR catalyzes the rate-limiting step in the biosynthesis of all four precursors for DNA replication, its activity is tightly regulated during the cell cycle, which generates a periodic fluctuation of the dNTP concentration in proliferating cells. As mentioned above, the enzyme’s allosteric specificity regulation controls the balanced concentrations of dNTPs. In mammalian cells, RNR activity in the G0/G1 phase is suppressed by transcriptional repression of the R2 gene and by anaphase-promoting complex Cdh1-dependent degradation of the R2 subunit in the M phase [57,58]. The enzyme activity and R1 and R2 mRNAs reach maximal levels during S phase [59,60,61]. The R1 subunit has a long half-life of ~18–24 h, and its protein levels are relatively constant and in excess throughout the cell cycle. The R2 protein has a shorter half-life of ~3–4 h and is specifically expressed during the S phase [60,61]. In addition to the conserved transcriptional repression mechanism, the RNR activity is also controlled by a small inhibitor protein in yeasts (Sml1 in *Saccharomyces cerevisiae* or Spd1 in *Schizosaccharomyces pombe*), that binds to RNR in the G1 phase [62,63]. The small inhibitor proteins are degraded upon entry into S phase or in response to DNA damage. Another regulation of RNR is achieved by differential cellular localization of its subunits during the cell cycle and after DNA damage or S phase arrest, and this regulation mechanism appears to be conserved among eukaryotic organisms [63,64,65,66,67]. In bacteria, the transcriptional regulation of RNR activity also plays a critical role during the cell division or under various growth conditions [68,69].

In the presence of HU, proliferating cells are arrested in S phase due to the decreased levels of dNTPs, which slows the DNA polymerase movement at replication forks. In eukaryotes, slowed forks activate the replication checkpoint, a highly conserved intracellular signaling pathway that is crucial for the maintenance of genome stability under replication stress [70,71]. The activated checkpoint stimulates RNR activity by increasing the production of R2, removing the small inhibitor proteins, and regulating the subcellular localization of R2. The activated checkpoint also delays mitosis, suppresses the firing of late origins, and stabilizes the slowed replication forks against collapse so that normal DNA synthesis can properly resume when the HU effect diminishes [72,73,74]. Without the checkpoint protection, the HU-treated forks are unstable and may undergo catastrophic collapse. Collapsed forks generate strand breaks and oxidative stress [22,75], which is generally believed to be the direct cause of cell death. Since the activated checkpoint delays cell division, mitotic catastrophe of the HU-treated cells lacking a functional checkpoint is likely another cause of the cell death [76,77]. Therefore, checkpoint mutants are highly sensitive to HU. However, cells with an intact checkpoint response are relatively insensitive to HU and the HU-induced S phase arrest is generally reversible in wild type cells after the drug removal [72,78].

Due to the reversible S phase arrest, HU is generally considered to be cytostatic, particularly to non-cycling cells [7,78,79,80]. However, earlier studies showed that at high concentrations or with prolonged exposure at lower doses, HU is cytotoxic to various mammalian cells such as Chinese hamster cells, mouse lymphoma cells, Ehrlich ascites tumor cells, and human lymphocytes [79,81,82,83,84], although HeLa and A549 lung carcinoma cells appear to be less sensitive [85,86]. Cytotoxicity after HU administration has also been found in rat and mouse proliferating tissues and embryos [87,88,89,90]. At high concentrations (more than 10 mM), HU is also cytotoxic to *E. coli.* Earlier studies showed that the cytotoxic effect of HU in both mammalian cells and *E. coli* appears to be linked to the accumulation of DNA strand breaks in HU-treated cells [91,92] or caused directly by reactive intermediates of HU that are generated in prolonged incubation [93,94,95,96]. A more recent report showed that, in vitro, HU can directly cause Cu(II)-mediated DNA damage particularly at thymine and cytosine residues, probably via the formation of H_2_O_2_ and nitric oxide [97]. However, whether HU induces DNA damage by itself or via its reactive derivatives in vivo remains unknown. Furthermore, since the checkpoint and the recently found sterol or heme biosynthesis mutants in *S. pombe* are highly sensitive to HU (see below) and the genetic backgrounds of the cell lines used in the earlier studies are unknown, the linkage between the DNA damage and the cell killing effect of HU may need to be reconsidered with caution.

## 4. Accumulation of Reactive Oxygen Species (ROS) 

ROS is a collective term used to describe ions and free radicals containing derivatives of molecular oxygen that are more reactive than oxygen itself. The ROS formed inside living cells commonly includes superoxide anion, hydrogen peroxide, and hydroxyl radical [98]. The normal process of respiration in mitochondria is a major source of endogenous ROS. Production of ROS is enhanced when mitochondrial function is perturbed or when the cells are under stress conditions. Accumulation of large amounts of ROS, particularly the deleterious hydroxyl radical, causes extensive oxidation of macromolecules, which directly contributes to cell killing.

To explain the mechanisms of rapid cell killing in the S phase and the teratogenic effect of HU, DeSesso hypothesized in 1979 that HU may exert its cytotoxic effects through radical chain reactions initiated by its hydroxylamine group, and predicted that antioxidants should ameliorate the cytotoxic and teratogenic effects of HU [18]. Subsequent studies by his group and other labs showed that radical scavengers substantially ameliorated the cytotoxic and teratogenic effects of HU [83,99,100]. These earlier studies suggest that accumulation of ROS might be involved in the cell-killing process of HU. More recent studies in *E. coli* using systems-level analyses have revealed the genomic and physiological effects of HU treatment that lead to cell death [19,20]. It was found that during the initial stage of HU treatment, several cell survival responses are activated, including upregulation of the SOS response, downregulation of cell division inhibition, and induction of the synthesis of RNR and the primosome components at the forks. As the HU treatment continues, the toxin modules MazF and RelE are activated, which trigger membrane stress and a cascade of events that eventually lead to the production of highly reactive hydroxyl radicals [101]. Production of hydroxyl radical is exacerbated by increased iron uptake, which promotes hydroxyl radical formation via Fenton chemistry [102]. An accumulation of harmful amounts of ROS is believed to contribute to the majority of HU-mediated cell death in *E. coli* [20,98]. Consistent with this notion, addition of the hydroxyl radical scavenger thiourea to the medium, suppresses HU sensitivity, and depletion of AphC, a component of the major scavenger enzyme of endogenous H_2_O_2_ alkyl hydroperoxide reductase [103], enhances HU sensitivity. Furthermore, deletion of genes involved in respiration and energy production, which decreases endogenous ROS production, confers resistance to HU [20]. Interestingly, elevated ROS levels and the resulting oxidation of guanine nucleotide pool has been shown to be a common mechanism that underlies cell death induced by all three major classes of bactericidal antibiotics [104,105].

Wild type yeasts such as *S. cerevisiae* and *S. pombe* are relatively insensitive to HU. However, recent studies suggest that HU treatment may generate ROS in both species. In addition to the DNA damage and environmental stress responses, HU treatment activates the Yap and Aft regulons in *S. cerevisiae* that function in redox and iron homeostasis respectively [24,106,107]. As a result, depletion of Yap1 moderately sensitizes the cells to HU, suggesting that ROS may be increased at an intermediate level or redundant factors exist in *S. cerevisiae* [24,106,107,108]. The Yap1 homologous protein in *S. pombe* is Pap1. Similar to that in *S. cerevisiae*, depletion of Pap1 also moderately sensitized *S. pombe* to HU [109], suggesting that HU treatment may generate oxidative stress in various eukaryotic organisms. Interestingly, overexpression or increased nuclear accumulation of Pap1 also confers the resistance on *S. pombe* to various other agents such as staurosporine [110], caffeine [111], and berefeldin A [112] and to DNA damage in checkpoint deficient mutants [113]. Scavenging the tyrosyl radical in RNR may also generate the hydroperoxy radical form of HU [17], which diffuses away and directly or indirectly modifies Yap1, leading to its accumulation inside the nucleus and transcriptional activation of genes involved in the redox response [106,114]. The activated Aft regulon promotes iron uptake, which may exacerbate the oxidative stress via Fenton reaction [19,115]. Consistent with these possibilities, overexpression of Yap1 can suppress the HU sensitivity caused by mutations in iron binding proteins such as Apd1 [108]. Apd1 is a thioredoxin-like ferredoxin protein. Mutation of the iron binding pocket or loss of Apd1 moderately sensitizes the cells to HU and the sensitivity can be rescued by antioxidant N-acetyl-cysteine [108].

The ROS generated by HU treatment can also alter the functions of proteins that contain iron–sulfur centers. For example, Dre2-Tah18 protein complex functions in cytosolic iron–sulfur protein biogenesis [116,117] and RNR metallocofactor assembly [118,119]. Mutation in Tah18 sensitizes *S. cerevisiae* to chronic treatment with HU [118,120] and overexpression of Yap1 can suppress the HU sensitivity caused by the Tah18 mutation [23]. Similar to that in *S. cerevisiae*, *E. coli* cells devoid of YfaE protein, which contains an iron–sulfur cluster and is required for the diferric tyrosyl radical cofactor maintenance of RNR, are also sensitive to HU [20,121].

Together, these studies show that HU may kill the cells by affecting the iron–sulfur clusters in proteins that function in the maintenance of the diferric tyrosyl radical center in RNRs or other cellular processes. Without a proper maintenance of diferric tyrosyl radical center in RNRs, the radical may leak into the cytoplasm and generate superoxide [20]. Interestingly, because iron–sulfur centers are sensitive to oxidative agents [122,123,124] and several eukaryotic replication proteins such as primase and Pol3 are known to contain iron–sulfur clusters [116,125,126], it is possible that oxidative stress generated by HU may directly suppress DNA replication. Although this mechanism of HU on DNA replication needs further investigation, it may provide an explanation to the replication arrest in the presence of basal dNTP levels that have been observed in HU-treated cells [23]. A recent study showed that HU could also trigger the accumulation of ROS in plant cells [127], suggesting that it is likely that this cell-killing mechanism of HU is highly conserved.

We have recently found in *S. pombe* that, similar to a previous study [22], the levels of ROS are only slightly increased in HU-treated wild type cells. However, in a *hem13* mutant, in which the heme level is low due to the hypomorphic mutation of the enzyme coproporphyrinogen III oxidase in the heme biosynthesis pathway, the levels of ROS as well as protein carbonylation, an indicator of the oxidation of various macromolecules, were significantly increased in HU-treated cells (our unpublished data). Unlike the checkpoint mutants that usually die within 2 to 3 h or one cell cycle time in HU, the *hem13* mutant is highly sensitive only to chronic treatment of HU. Similar chronic HU sensitivity was also observed in *S. cerevisiae* lacking Sod1, the enzyme that catalyzes the decomposition of superoxide [128], which suggests that the cell killing caused by HU-induced oxidative stress is a slow process. Furthermore, the HU sensitivity of the *hem13* mutant can be suppressed by culturing the cells under anaerobic conditions, which inhibits aerobiosis and thus decreases the production of endogenous ROS. Like the *S. cerevisiae* cells lacking Sod1, increased RNR activity cannot rescue the HU sensitivity of the *hem13* mutant, which is consistent with the notion that the *hem13* mutant is killed by a mechanism that is unrelated to dNTP depletion.

## 5. Cytokinesis Arrest and the Potentially Unidentified Cellular Target(s) of HU

**Figure 2:**
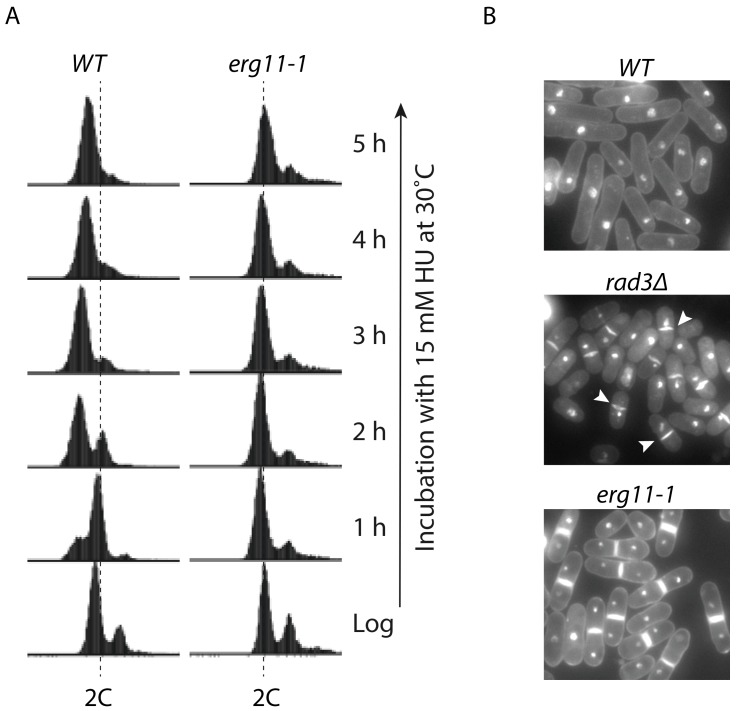
HU induces cytokinesis arrest in *Schizosaccharomyces pombe erg11-1* mutant. (**A**) unlike wild type (WT) cells that are arrested in S phase, HU arrests *erg11-1* cells in G2/M phase. Cell cycle progression of the wild type and *erg11-1* mutant cells cultured in YE6S medium containing 15 mM HU was monitored during the course of incubation at the indicated time points by flow cytometry. Dashed lines indicate the cells with a 2C DNA content. Since most of the *S. pombe* cell cycle time is at G2 phase, the majority of the logarithmically growing cells (Log) have a 2C DNA content; (**B**) wild type *S. pombe*, the checkpoint mutant *rad3∆* lacking the sensor protein kinase Rad3 (ortholog of human ATR and *Saccharomyces cerevisiae* Mec1), and *erg11-1* cells were treated with 15 mM HU for 3 h at 30 °C in YE6S medium and then stained with propidium iodide (PI) for genomic DNA and Blankophor for cell wall and the septum. The stained cells were examined under a fluorescent microscope. Arrowheads indicate cells with the “cell untimely torn” or *cut* phenotype in *rad3∆* cells, a strong indicator of aberrant mitosis in HU-treated checkpoint deficient mutants [77]. (This figure is adapted from the reference [21] with permission from The Genetics Society of America).

While screening for new mutants in *S. pombe* that are sensitive to replication stress, we identified a new hypomorphic mutation, *erg11-1*, that dramatically sensitizes the cells to chronic, but not acute treatment with HU [21] (Figure 2). The gene product of *erg11* is the enzyme sterol-14α-demethylase, which is required for ergosterol biosynthesis and a major target of antifungal agents. We found that, unlike wild type cells that are arrested in S phase, HU arrests the mutant cells mainly in cytokinesis. The HU-induced cytokinesis arrest is relatively stable and occurs at low doses of HU, which likely explains the remarkable HU sensitivity. HU hypersensitivity has also been observed in several *erg* mutants in *S. cerevisiae*, including *erg10-1,* which encodes the first enzyme in the ergosterol biosynthesis pathway acetolacetyl-CoA thiolase [129], and *erg3* that encodes C-5 sterol desaturase [130]. Although the underlying mechanism of the HU sensitivity in these *S. cerevisiae* mutants remains to be determined, HU may suppress cell division in the presence of sterol deficiency in diverse eukaryotic organisms.

**Figure 3:**
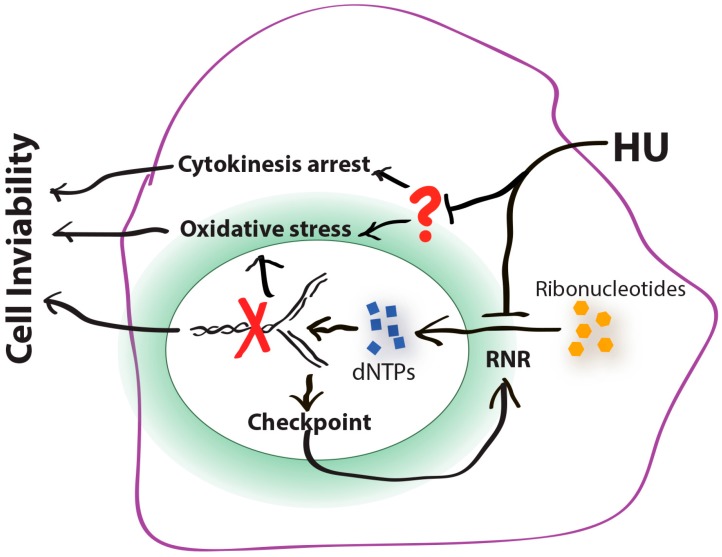
The cell-killing mechanisms of HU. HU inhibits its primary cellular target ribonucleotide reductase (RNR), which decreases the deoxyribonucleotide triphosphate (dNTP) levels and slows the movement of DNA polymerases at the forks (**red** cross). Slowed forks activate the DNA replication checkpoint. Activated checkpoint stimulates RNR to increase the dNTP production for DNA synthesis and fork recovery. Activated checkpoint can also suppress mitosis to prevent aberrant cell division (not shown). Without a functional checkpoint, slowed forks collapse and thus generate DNA damage, which leads to cell inviability. Recent studies suggest that, in addition to RNR, HU may have a secondary target(s) (**red** question mark) such as the metal enzymes and the matrix proteases that have been reported recently [52,53,54]. Suppression of the secondary target(s) may arrest the cells in cytokinesis or generate oxidative stress, which also leads to cell lethality. In *Escherichia coli*, oxidative stress is the common mechanism underlying the cell killing process of all three major classes of bactericidal antibiotics [105]. It has been shown that fork collapse generates oxidative stress in yeast [22]. Whether the HU-induced cytokinesis arrest also generates oxidative stress in eukaryotes remains to be investigated.

Since the cytokinesis arrest occurs at HU concentrations much lower (1–3 mM) than that required for replication arrest (more than 6 mM) in *S. pombe*, it is possible that, in addition to RNR, HU may have a secondary target(s) involved in cell division that can be unmasked by sterol deficiency and become druggable to HU (Figure 3). As mentioned above, HU has been reported to inhibit catalase and several metalloproteases. It is possible that sterol deficiency may synergize with HU in suppressing the secondary target(s) and thus arrest the cells in cytokinesis. In support of this hypothesis, various combinations of HU and sterol synthesis inhibitors have shown synergistic antifungal effects (our unpublished data). Since almost all of HU-treated *erg11-1 S. pombe* contains two nuclei and a brightly stained septum that is well positioned in the middle of the cells, the arrest may be caused by a defect in the late stage of cytokinesis. Several *S. pombe* mutants have been reported that show the similar late stage cytokinesis arrest [131]. Genetic studies by crossing the *erg11-1* mutant with cytokinesis mutants such as *byr4*, *cdc16*, *dma1* and *nuc2,* may pinpoint the exact step where the arrest occurs and thus the identification of the target(s) of HU [132,133,134,135]. Cytokinesis is the last step of the cell division cycle that is crucial for cell proliferation. It has been extensively exploited for the development of anti-neoplastic chemotherapeutics [136,137]. Identification of the secondary target(s) of HU in cytokinesis may therefore help to develop new therapeutics for the treatment of cancers or infectious diseases.

## 6. Conclusions

This review has focused upon the mechanisms by which HU exerts its cytotoxic effects. Clearly our knowledge is far from complete. For example, how are ROS generated in HU-treated cells? Fork collapse can clearly generate ROS [19,20,22]. However, the exact mechanism by which fork collapse causes ROS accumulation remains to be determined. Because some of the HU hypersensitive yeast mutants are killed at drug concentrations significantly lower than that required for slowing down the fork progression, ROS have to be accumulated via a different mechanism. In addition, the cytokinesis arrest observed in the *S. pombe erg11-1* mutant is clearly caused by a previously unknown mechanism [21] and is consistent with the existence of the secondary unknown target(s) in eukaryotic organisms [52,53,54,55] (Figure 3). Since HU has been used for the treatment of various cancers and infectious diseases, identification of such targets and characterization of the new cell-killing mechanisms of HU, particularly in the non-proliferating cells, may provide new strategies for improving the HU-based chemotherapies [45,56].

## Figures and Tables

**Table 1:** List of potentially new targets of hydroxyurea (HU) that have been discovered recently.

Potential Targets	Discovery Methods	Organisms	Biological Functions	Ref.
Catalase	Genetics	*A. thaliana*	Decomposition of H_2_O_2_	[52]
Carbonic anhydrase	in vitro	?	Interconversion of CO_2_ and H_2_O to H_2_CO_3_	[53]
Matrix metalloproteinases	in vitro	?	Cleavage of the peptide bond	[54]
Unknown yet	Genetics	*S. pombe*	Cytokinesis	[21]
